# Electrocatalyst of PdNi Particles on Carbon Black for Hydrogen Oxidation Reaction in Alkaline Membrane Fuel Cell

**DOI:** 10.3390/nano15090664

**Published:** 2025-04-27

**Authors:** Carolina Silva-Carrillo, Edgar Alonso Reynoso-Soto, Ivan Cruz-Reyes, Moisés Israel Salazar-Gastélum, Balter Trujillo-Navarrete, Sergio Pérez-Sicairos, José Roberto Flores-Hernández, Tatiana Romero-Castañón, Francisco Paraguay-Delgado, Rosa María Félix-Navarro

**Affiliations:** 1 Tecnológico Nacional de México, Instituto Tecnológico de Tijuana, Centro de Graduados e Investigación en Química, Blvd. Alberto Limón Padilla S/N, Mesa de Otay, Tijuana C.P. 22500, Baja California, Mexico; carolina.silva.carrillo@uabc.edu.mx (C.S.-C.); edgar.reynoso@tectijuana.edu.mx (E.A.R.-S.); ivan.cruz19@tectijuana.edu.mx (I.C.-R.); moises.salazar@tectijuana.edu.mx (M.I.S.-G.); balter.trujillo@tectijuana.edu.mx (B.T.-N.); sperez@tectijuana.mx (S.P.-S.); 2 Instituto Nacional de Electricidad y Energías Limpias (INEEL), Ave. Reforma 113, Col. Palmira, Cuernavaca C.P. 62490, Morelos, Mexico; jrflores@ineel.mx (J.R.F.-H.); tromero@ineel.mx (T.R.-C.); 3 Centro de Investigación en Materiales Avanzados (CIMAV), Av. Miguel de Cervantes #120, Complejo Industrial Chihuahua, Chihuahua C.P. 31136, Chih, Mexico; francisco.paraguay@cimav.edu.mx

**Keywords:** bifunctionality, PdNi, carbon Vulcan, HOR, anode, AEMFC

## Abstract

This work reports the synthesis of PdNi bimetallic particles and Pd on Carbon black (Vulcan XC-72) by reverse microemulsion and the chemical reduction of metallic complexes. The physicochemical characterization techniques used for the bimetallic and metallic materials were TGA, STEM, ICP-OES, and XRD. Also, the electrocatalysts were studied by electrochemical techniques such as anodic CO stripping and β-NiOOH reduction to elucidate the Pd and Ni surface sites participation in the reactions. The electrocatalysts were evaluated in the anodic reaction in anion-exchange membrane fuel cells (AEMFC) and the hydrogen oxidation reaction (HOR) in alkaline media. The results indicate that PdNi/C electrocatalysts exhibited higher electrocatalytic activity than Pd/C electrocatalysts in both the half-cell test and in the AEMFC, even with the same Pd loading, which is attributed to the bifunctional mechanism that provides OH^-^ groups in oxophilic sites associated to Ni, that can facilitate the desorption of Hads in the Pd sites for the bimetallic material.

## 1. Introduction

The problem of managing CO_2_ emissions in industrialized countries has promoted the development of several renewable energies [1,2]. Unfortunately, most of the time, the intermittence of renewable prevents the integration of those technologies into the electrical energy network that brings service to the population. This drawback of renewable energies can vanish by implementing energy conversion/storage devices integrated into the grid energy during the deficit of electrical energy and saving energy during the surplus of electrical energy [3]. Hydrogen is widely regarded as a highly advantageous energy source, primarily because of its substantial energy density and its capacity to produce electricity without harmful emissions. Its efficiency as a fuel stems from its impressive thermal energy value, approximately 120 MJ/kg, making it superior to other hydrocarbons [4]. At present, hydrogen is pivotal in numerous industrial processes and applications, including fuel cell technologies [5], ammonia synthesis [6], and the catalytic hydrogenation of unsaturated compounds [7,8].

Fuel cells are advanced electrochemical devices designed to produce clean electrical energy, boasting a theoretical efficiency of over 80% in continuous operation. This high-efficiency position of fuel cells stands out as a crucial component of pivotal technologies for energy conversion [2]. Various categories of commercial fuel cells are characterized by the fuels utilized, the catalysts applied, and the electrolytes employed. Among them, polymeric electrolyte fuel cell technology has drawn considerable attention from the scientific community due to its straightforward operation and low-temperature requirements. This category is further divided into proton exchange membrane fuel cells (PEMFC) and anionic exchange membrane fuel cells (AEMFC). AEMFCs offer notable advantages over PEMFCs, as the electrochemical kinetics of the oxygen reduction reaction (ORR) is more rapid under alkaline conditions, and they allow the use of non-noble metal electrocatalysts, making them a more cost-effective technology [9,10]. In AEMFCs, the key processes taking place at the cathode and anode consist of ORR and the hydrogen oxidation reaction (HOR), respectively. Unlike in PEMFCs, the HOR kinetics in AEMFCs are slower, which remains a limiting factor for their development, even with the application of Pt catalysts [11].

The lower kinetic rate can be explained in terms of the HOR mechanism, which is described as a process with successive steps, with the initial stage requiring the dissociative adsorption of hydrogen on the active sites (Equation (1)), called the Tafel step. Then, the next step involves the electron charge transference via either Heyrovsky or Volmer step (Equation (2) and Equation (3), respectively). Both steps involve the OH^−^, which can be taken from the media (outer sphere complex) or from another less noble metal (inner sphere complex):

(1) $$H_{2}+2M\vec{\leftarrow }2M-H_{ads}$$

(2) $$H_{2}+{OH}^{-}+M\vec{\leftarrow }M-H_{ads}+H_{2}O+e^{-}$$

(3) $$M-H_{ads}+{OH}^{-}\vec{\leftarrow }M+H_{2}O+e^{-}$$

According to the study by Markovic [12] the HOR kinetics in alkaline environments are constrained by the Volmer step. They suggest that reactive hydroxyl species (OH_ads_) are essential to accelerate this step. Therefore, using an oxophilic catalyst could supply OH_ads_ species, aiding in the restoration of the M sites on the hydrophilic catalyst.

Platinum group electrocatalysts (PGM) have been demonstrated to be the most widely used catalysts as anodes in AEMFC. Pd shows a lower oxophilic capacity than Pt [13] and exhibits a hydrogen bond energy (HBE) close to Pt [14]. In addition, the activity of the electrocatalyst in the HOR exhibits enhanced kinetics with increasing particle dimensions [15]. Pd electrocatalysts have shown low electrocatalytic activity for HOR compared to Pt electrocatalysts since d orbitals are fully occupied and have a low oxophilicity [14,16]. Adding a second metal has significantly improved the electrocatalytic activity of Pd-based materials. A catalytic activity-enhancing effect in HOR is due to the incorporation of an oxophilic metal and a hydrophilic metal in an electrocatalyst since one of the rate-determining steps of HOR is the hydrogen release of hydrogen species from the hydrophilic metal surface. In facilitating this process, the effect of a hydrophilic and oxophilic metal on its catalytic properties has been interpreted as a “bifunctional” activity [17].

Nickel (Ni), classified as an oxophilic metal, stands out as a highly promising candidate for catalyzing AEMFC anodes. This cost-effective metal exhibits remarkable corrosion resistance in alkaline conditions and ranks among the top non-precious metals in electrocatalytic activity for HOR within alkaline media [18,19].

Trasatti [20] compiled experimental findings related to the hydrogen reaction and introduced the pioneering volcano plot, which illustrates the correlation between exchange current density and M-H bond energy. While platinum (Pt) and other noble metals occupy the apex of the plot, nickel (Ni) exhibits a decrease in M-H bond energy—an essential factor associated with adsorption and desorption processes [21]. Moreover, Ni not only reduces M-H bond energy but also facilitates the formation of OH_ads_ species, thereby enhancing catalytic efficiency.

Despite these advantages, the literature on Ni-based catalysts for hydrogen oxidation reaction (HOR) applications remains scarce. Kiros [22] reported the synthesis of Raney Ni modified with other transition metals. Their results revealed limited HOR activity, attributed to an insufficient ratio between the active surface area and catalyst mass. Despite extensive research efforts, the mass-normalized electrocatalytic performance of Ni-based catalysts in HOR continues to lag significantly behind that of noble metal catalysts.

Savinova [23,24] demonstrated that the incorporation of a second metal enhances catalytic activity by decreasing hydrogen binding energy. Additionally, studies have examined the impact of metal atomic distribution on HOR performance in PdNi bimetallic nanoparticles. The proposed bifunctional catalytic mechanism suggests that hydrogen adsorption occurs at Pd sites while OH adsorption takes place at Ni sites. Consequently, PdNi nanoparticles exhibit mass activities approximately 13 times greater than those of pure Pd nanoparticles [25].

The bifunctional nature of electrocatalysts has been demonstrated in PdNi systems. PdNi catalysts synthesized via a colloidal chemical method [15] showed superior HOR onset potential in alkaline conditions compared to pure Pd, with a positive shift of approximately 200 mV. The evaluation of this material was conducted in a membrane electrode assembly (MEA) designed for an AEMFC, resulting in a peak power output of 400 mW cm^−2^, which is more than twice the maximum power achieved with monometallic Pd electrocatalysts. This improved performance arose from the interaction between Pd and Ni nano-islands, wherein OHads species modulated the surface coverage of Hads on Ni, thereby enhancing HOR kinetics. Truong [26] describe the use of PdNi/C anodic electrocatalysts in AEMFC applications. PdNi nanoparticles supported on carbon black, with varying Pd:Ni weight ratios, reached a power output of 302 mW cm^−2^ and exhibited higher current density for a 50:50 wt.% Pd:Ni ratio.

There are scarce reports using PdNi bimetallic electrocatalysts as anode in AEMFC. Most of the reports focus on studying the electrocatalytic activity in a half cell. In the few reported works, they use high Ni loadings ratios compared to Pd to reach high powers and metal charges of 1.5 mg cm^−2^ and large areas of the active electrode to improve the efficiency [16,26].

In this work, PdNi/C showed a great exposure of both Ni and Pd active sites in the bimetallic material, generating a great electrocatalytic activity in HOR. The catalyst was synthetized with low metallic loadings, and the performance at the HOR was studied, obtaining higher current density compared with Pd/C. Finally, the PdNi/C catalyst was used to fabricate the AEMFC anode with a catalyst loading of 0.5 mg cm^−2^ and a Pd:Ni weight ratio of 2:1 in the carbon-supported PdNi electrocatalyst. The resulting PdNi/C demonstrated a power density three times greater than that of Pd/C, showcasing the exceptional catalytic activity of this bimetallic system. This research signifies a notable step forward in the field, demonstrating superior catalytic performance with low metal loadings and tackling both efficiency and economic feasibility in AEMFC applications.

## 2. Materials and Methods

### 2.1. Reagents

All chemicals used in this study were employed without further purification. For the experiments the following reagents were purchased from by Merck (Darmstadt, Germany): nickel(II) acetate tetrahydrate (Ni(OCOCH_3_)_2_·4H_2_O, 98%), cetyltrimethylammonium bromide (CH_3_(CH_2_)15N(Br)(CH_3_)_3_, ≥98%), sodium borohydride (NaBH_4_, 98%), sodium citrate tribasic dehydrate (HOC(COONa)(CH_2_COONa)_2_·2H_2_O, 99%), sodium tetrachloropalladate(II) (Na_2_PdCl_4_, 98%), Nafion^®^ 117 (5 wt%). The nitric acid (HNO_3_, ACS ≥ 70%), isopropanol (C_3_H_8_O, 99%), ethanol (C_2_H_5_OH, 99%), methanol (CH_3_OH, 98%), were purchased from Fermont (Mexico City, Mexico).

### 2.2. Synthesis of Ni/C, PdNi/C and Pd/C

**Scheme 1:**
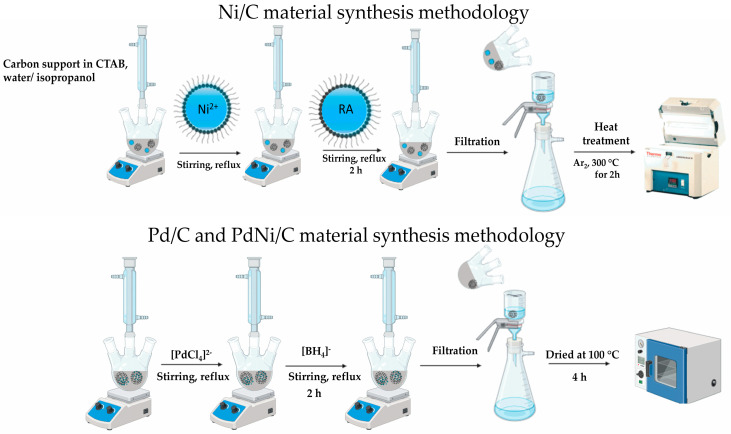
Synthesis scheme methodology for Ni/C, Pd/C, and PdNi/C materials.

To prepare the nickel template supported on carbon (Ni/C), the carbon material was subjected to acid treatment using a 0.5 M HNO_3_ solution under reflux and constant stirring for 30 min. The resulting dispersion was subjected to centrifugal separation at 4000 rpm, then extensively rinsed with deionized water until the supernatant reached a neutral pH of 7. Scheme 1 shows the synthesis scheme methodology for Ni/C, Pd/C, and PdNi/C.

The fabrication of the Ni/C template was executed through a dual-step procedure using the reverse microemulsion method, as previously described [27]. Initially, the carbon material was dispersed in a microemulsion consisting of 4 g of CTAB dissolved in an isopropanol/water mixture (87/13% *v*/*v*). The dispersion was then heated under reflux, and sequentially, a microemulsion containing reducing agents (RA), 0.04 mmol of Ni(OCOCH_3_)_2_·4H_2_O was introduced, followed by another microemulsion with 0.20 mmol of NaBH_4_ and 0.17 mmol of C_6_H_5_Na_3_O_7_. The reaction system was maintained under continuous agitation continuously for 2 h. Subsequently, the dispersion Underwent filtration was followed by washing with 2-propanol, water, and acetone to remove residual solids. Finally, thermally processed at 300 °C for a duration of 30 min in a tubular furnace was applied to eliminate the surfactant and by-products from the synthesis process.

For the synthesis of bimetallic PdNi/C or monometallic Pd/C electrocatalysts, direct palladium reduction was performed on either the Ni/C template or the carbon. The Ni/C or The carbon was evenly distributed in a methanol solution, and a palladium precursor solution was added. The dispersion was heated under reflux, and a methanolic solution of NaBH_4_ was gradually introduced. The reaction was maintained under constant stirring for 2 h. After completion, the mixture underwent filtration underwent rigorous purification with methanol, water, and acetone and was subsequently subjected to drying at 100 °C for 4 h.

### 2.3. Physicochemical Characterization

The thermal characteristics and elemental composition of the materials were assessed via thermogravimetric evaluation (TGA) performed on a TA Instruments Q500 system (TA Universal Analysis 2000 Software, New Castle, DE, USA). Thermal profiles were acquired across a temperature interval spanning 20 °C to 850 °C, applying a heating rate of 20 °C per minute within a regulated dry air environment. The metallic constituents were analyzed using inductively coupled plasma optical emission spectroscopy (ICP-OES) employing a Perkin-Elmer Optima 8300 instrument (Syngistix Software, Waltham, MA, USA). Solid samples of 5 mg were subjected to calcination, and the resulting residue was subsequently dissolved in aqua regia. The operational parameters for the gas nebulizer and auxiliary flow were adjusted to 15, 0.2, and 0.55 L min^−1^, respectively. Spectroscopic measurements were conducted in axial mode under a radiofrequency power of 1300 W. A sample volume of 1 mL was utilized for analysis, with selected detection wavelengths of 340.5 nm for Pd and 231.6 nm for Ni. Each sample underwent triplicate measurements to ensure reproducibility.

The structural characteristics and shape of Pd nanoparticles (NPs) were examined utilizing an advanced transmission electron microscope JEM-2200FS, JEOL (Tokyo, Japan) operating at 200 kV. This instrument is equipped with spherical aberration correction, enabling a spatial resolution of 0.1 nm in STEM mode. Additionally, X-ray diffraction (XRD) measurements were conducted using a D8 Advance diffractometer, Bruker brand (Billerica, MA, USA) with Cu Kα radiation (λ = 1.541 nm). The scanning process was performed at a rate of 0.01° s^−1^, covering a 2θ angular range from 10° to 80°.

### 2.4. Electrochemical Characterization and Electrocatalytic Activity for HOR

The investigation of the electrochemical properties and electrocatalytic activity for HOR was performed using a Biologic SP-150 potentiostat/galvanostat (EC-Lab^®^, Nashville, TN, USA). The experiments employed a setup with three electrodes at a controlled temperature of 20 ± 1 °C, consisting of a glassy carbon substrate functioning as the primary electrode, an Hg/HgO/NaOH (1 M) system as the reference, and a platinum wire serving as the auxiliary electrode. Catalytic inks were formulated by mixing 2 mg of either monometallic or bimetallic electrocatalysts in an ethanol/Nafion^®^ 117 solution (1:3 *v*/*v*) (Merck, Darmstadt, Germany). Electrochemical evaluations took place in a 0.1 M KOH electrolyte.

The determination of the electrochemical surface area (ECSA) utilized a glassy carbon substrate with a surface area of 0.079 cm^2^, modified with 20 microliters of the catalyst ink. The ECSA was calculated through two separate approaches [27,28]. For palladium (Pd), CO stripping cyclic voltammetry was conducted across a potential window ranging from 0 to 1.3 V relative to RHE at a scan rate of 50 mV/s in an Ar-saturated solution. After saturating the electrolyte with CO, a potential of 0.05 V relative to RHE was maintained for 15 min, followed by recording a polarization curve within the same potential interval at a speed of 100 mV/s. For nickel (Ni), the β-NiOOH reduction technique was implemented, involving pretreatment of the Ni sites in the electrocatalyst by applying a steady potential of −0.08 V relative to RHE, succeeded by a polarization curve recorded between 0.10 and 0.23 V relative to RHE at a scan rate of 100 mV/s.

For the HOR analysis, the main electrode was a glassy carbon disk (0.196 cm^2^ geometric area) modified with 40 microliters of the catalyst ink. Initially, 30 voltammetry cycles were performed in 0.1 M KOH saturated with nitrogen over a potential interval spanning from −0.1 to 1.5 V relative to RHE at a scan rate of 100 mV/s. Subsequently, HOR polarization curves were acquired in a hydrogen-saturated 0.1 M KOH electrolyte by progressively increasing the potential from 0 to 1.0 V relative to RHE at a rate of 10 mV/s under various rotational speeds.

### 2.5. AEMFC Performance Test

AEMFC performance evaluation was carried out using a custom-designed single-fuel cell setup. The anode utilized Pd/C and PdNi/C electrocatalysts, while the cathode incorporated commercial Pt/C (20 wt%) in all tests. Membrane electrode assemblies (MEAs) were formed by spraying catalyst ink onto gas diffusion layers (GDLs). The ink consisted of the catalyst dispersed in a mixture of water, AEMION+^®^ inomer825 (Vancouver, BC Canada), and ethanol. The metal loading on the GDLs was set at 0.5 mg cm^−2^, with an active surface area of 5 cm^2^. A commercial AEMION+^®^ AF1-HNN8-25 membrane with a thickness of 50 µm served as the separator between the cathode and anode. Prior to assembling the cell, both the membrane and GDLs were hydrated for 24 h, followed by a hydroxyl ion exchange process in 1.0 M KOH for 48 h.

Fuel cell tests were performed using the Scribner Fuel Cell Test System, model 850e. Oxygen and hydrogen were supplied to the cathodic and anodic compartments, respectively, at flow rates of 800 and 400 mL min^−1^, with dew points of 57 °C and 54 °C. Throughout the tests, the cell temperature remained constant at 60 °C with a back pressure of 10 psi. Potentiodynamic polarization measurements were conducted by sweeping the potential from the open-circuit potential (OCP) to 0.3 V at a scan rate of 2 mV s^−1^. Each sample underwent duplicate tests. Electrochemical impedance spectroscopy (EIS) was employed to determine ohmic resistance, with frequency ranges from 100 kHz to 0.1 Hz. Measurements were taken with a 10 mV amplitude at 0 V DC using 10 steps per decade.

## 3. Results and Discussion

### 3.1. Physicochemical Characterization

**Figure 1:**
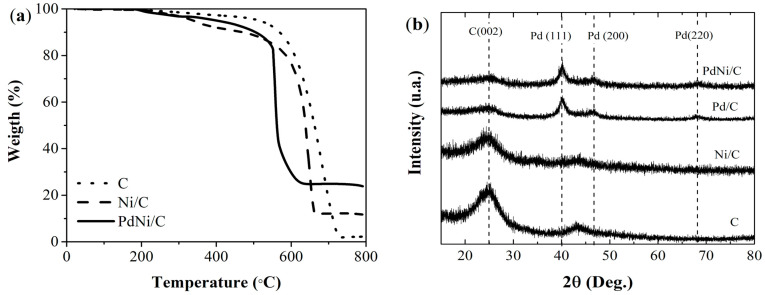
Thermograms (**a**) and diffraction patterns (**b**) of C support Ni/C, Pd/C, and PdNi/C undecorated.

Figure 1a shows the thermogravimetric curves of the undecorated carbon supports (C) and those decorated with monometallic (Ni/C) and bimetallic particles (PdNi/C). All materials exhibit three stages of decomposition. For the undecorated C, the first stage of decomposition occurs between 150 and 276 °C, corresponding to the breakdown of functional groups formed during the acid treatment of the carbon support. The second phase represents the degradation of amorphous carbon, occurring between 305 and 510 °C for C. Finally, the disintegration of the remaining carbon support is observed between 536 and 735 °C of CV. At temperatures exceeding 735 °C, a stable percentage remains in both carbon supports, attributed to residual metal oxides. When incorporating Ni and subsequently Pd, an increase in the residual metal oxide percentage is observed (Table 1).

The metallic content data of the materials obtained by TGA and ICP-OES analysis are shown in Table 1. The weight percentages of Ni and Pd in the metallic and bimetallic catalysts are similar, with approximately a Ni/Pd ratio of 1:2 *w*/*w*.

The characterization of the synthesized materials through XRD analysis is shown in Figure 1b. For the C support, diffraction patterns at 25.0° and 26.2° of 2θ are evident, corresponding to the reflection plane (002), along with a peak at 43.8°, which relates to the graphitic planes of hexagonal carbon (004). The Pd/C and PdNi/C materials exhibited a peak at 25.0° of 2θ, corresponding to the C (002) plane of the carbon support. Additional peaks were observed at 40.0° and 47.1°, associated with the crystalline planes of Pd (111) and Pd (200). The Pd/C and PdNi/C materials also show a peak at 68.2°, attributed to the crystalline plane of Pd (220). These palladium crystalline planes correspond to the FCC (face-centered cubic) structure, as reported in previous studies [26,29]. The bimetallic material displays a larger Pd crystal size (calculated using the Scherrer equation) compared to the monometallic material (Table 1).

The BET areas presented in Table 1 were obtained from BET analyses shown in Figure S1. A reduction in surface area is observed when carbon is modified with monometallic and bimetallic nanoparticles. This phenomenon is attributed to the deposition of nanoparticles, which may occupy the pores within the carbon [30]. The SEM micrographs and EDS mapping in Supporting Information Figure S2 show the presence of metallic particles of Pd and Ni, uniformly distributed throughout the carbon supports.

**Figure 2:**
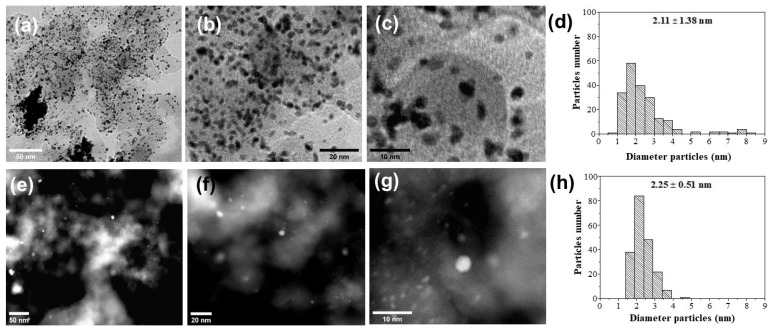
STEM images and histograms of materials PdNi/C (**a**–**d**) and Pd/C (**e**–**h**).

The uniform and well-distributed dispersion of PdNi particles across the surface of the C support is evident in the micrographs presented in Figure 2a–c, although some particle agglomerations can still be observed. For the Pd/C material, small particles with limited uniformity are visible, and the formation of clusters in various regions of the carbon support is noticeable. The mean particle size was determined through statistical analysis, as depicted in the inset (6d and 6h), and measured 2.11 ± 1.38 nm and 2.26 ± 0.61 nm for PdNi/C and Pd/C, respectively. The incorporation of nickel into the system does not significantly alter particle size; instead, nickel enhances the dispersion of nanoparticles along the support.

### 3.2. Electrochemical Characterization

**Figure 3:**
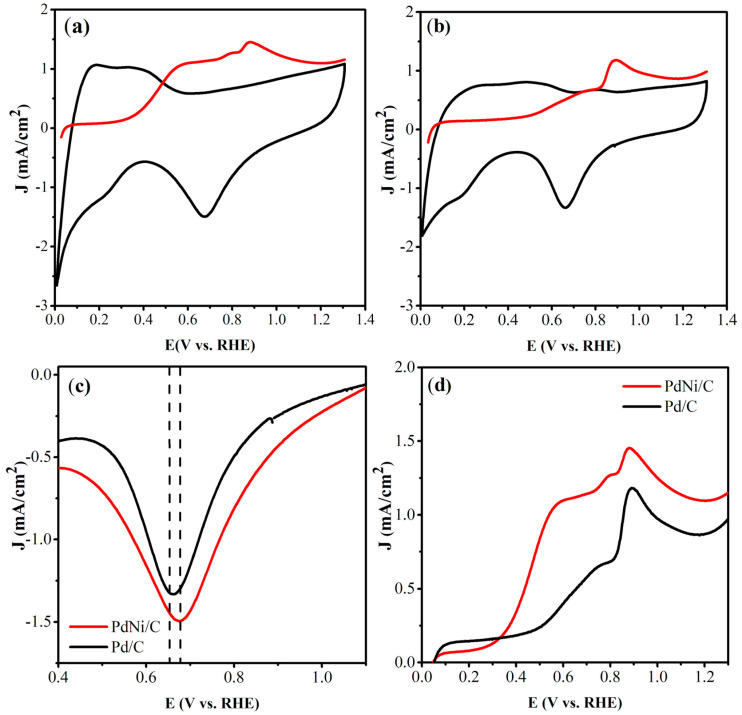
Cyclic voltammetry by CO stripping PdNi/C (**a**), Pd/C. (**b**) Comparison of the zone of reduction of Pd-OH_ads_ for PdNi/C and Pd/C electrocatalyst (**c**) and comparison of the oxidation of CO_ads_ for PdNi/C and Pd/C electrocatalyst (**d**).

The estimation of the ECSA of the monometallic (Pd/C) and bimetallic (PdNi/C) electrocatalysts was assessed using the techniques of anodic stripping of CO (ECSAPd) and reduction of β-NiOO (ECSANi). Figure 3a and Figure 3b display the voltammograms obtained using the anodic stripping of CO for the PdNi/C and Pd/C electrocatalysts, respectively. Voltammograms recorded without CO show variations in cathodic and anodic currents at lower potentials, corresponding to the processes of hydrogen adsorption and desorption on the Pd surface. At higher potentials (>0.69 V vs. RHE), an increase in anodic current is linked to OH- adsorption on both Pd and Ni surfaces. The OH- desorption signal appears around 0.67 V vs. RHE. The voltammogram recorded in the presence of CO reveals an anodic signal above 0.88 V vs. RHE (red line). These signals correspond to the oxidation of CO, as described in Equation (4) [27,29]:

(4) $${CO}_{ads}+{OH}_{ads}+{OH}^{-}\to {CO}_{2}+H_{2}O+e^{-}$$

The OH^−^ adsorption zones show a considerable rise in current density along with a shift of the potential peak towards less negative values when Ni is incorporated into the electrocatalyst, indicating an increase in its oxophilic properties. Figure 3c shows the zone of reduction of Pd-OH_ads_. The PdNi/C electrocatalyst shows a shift from the maximum desorption peak of Pd-OH_ads_ to less negative potential than the Pd/C electrocatalyst. This shift indicates that the bimetallic PdNi/C electrocatalyst has a weaker binding strength with the OH^−^ species than the monometallic Pd/C, while higher current density is related to higher OH_ads_ species on the catalyst surface [31].

Figure 3d illustrates the CO desorption region. The Pd/C electrocatalyst exhibits two distinct CO desorption zones on Pd sites, which can be linked to variations in the binding strength of CO molecules, with weak adsorption occurring at approximately 0.70 V and strong adsorption around 0.91 V [32]. For the bimetallic PdNi/C electrocatalyst, a single CO oxidation peak is detected at 0.89 V. The presence of Ni in bimetallic PdNi/C electrocatalyst makes Pd less poisoned by CO_ads_, as the oxidation of COads is accelerated by the presence of Ni, which can lead to lower oxygen-containing species through bifunctional catalytic mechanisms [33]. Furthermore, the increasing oxophilic feature enhances CO desorption, providing OH_ads_ species. Furthermore, a slight displacement of the anodic potential peak is observed for the PdNi/C electrocatalyst, indicating lower activation energy for the CO oxidation due to (i) OH_ads_ availability because the oxophilic nature of the bimetallic catalyst is changing and/or (ii) because a change in the electronic properties of the bimetallic electrocatalyst is taking place.

The estimation of ECSA_Pd_ is related to the area of the anodic peak by the desorption of CO_ads_ by using Equation (5) [33,34]:

(5) ECSAPd=QCO0.420 mCcm2LPd

where Q_CO_ represents the integrated area under the anodic peak resulting from CO desorption, the specific charge density amounts to 420 μC cm^−2^, and L_Pd_ denotes the Pd mass loading. The ECSA_Pd_ values are detailed in Table 2. The PdNi/C electrocatalyst demonstrated a higher ECSA_Pd_ value compared to Pd/C, suggesting that a greater number of Pd sites are accessible in the bimetallic electrocatalyst.

**Figure 4:**
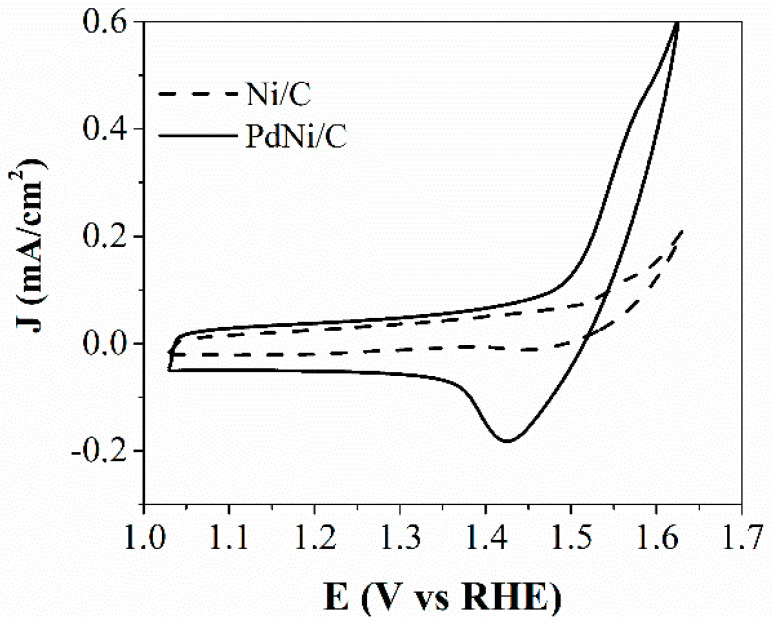
Cyclic voltammetry by the β-NiOOH technique for Ni/C and PdNi/C.

In evaluating the electrochemically active area related to Ni (ECSA_Ni_) in monometallic Ni/C and bimetallic PdNi/C electrocatalysts, the cyclic voltammograms corresponding to β-NiOOH reduction are depicted in Figure 4. The voltammogram reveals variations in both anodic and cathodic currents at higher potentials for the bimetallic PdNi/C electrocatalysts, whereas monometallic Ni/C electrocatalyst displays solely anodic current, linked to the redox reaction of β-Ni(OH)_2_, as defined by Equation (6) [27,28]:

Ni(OH)_2_ + OH^−^↔ β-NiOOH + e^−^ + H_2_O (6)

Because the shape of the anodic peak is not so obvious, the cathodic peak is used to calculate the ECSA_Ni_ through Equation (7) [27,28]

(7) ECSANi=Qβ−NiOOH0.420 mCcm2LNi

where Q_β-NiOOH_ corresponds to the integrated region beneath the reduction curve of β-NiOOH, L_Ni_ represents the Ni mass loading, and the charge density is specifically calculated at 420 μC cm^−2^. Table 2 displays the ECSA_Ni_ values of the monometallic Ni/C and bimetallic PdNi/C electrocatalysts. The PdNi/C exhibited a greater value compared to Ni/C. Additionally, bimetallic PdNi/C electrocatalysts demonstrate an ECSA_Ni_ value that surpasses that of monometallic Ni/C electrocatalysts by over 3.8 times.

The addition of a secondary metal enhances the adsorption and desorption mechanism for various species, reducing the adsorption energy associated with these species and increasing the number of active sites available. The results demonstrate that the ECSA aligns with the XRD and STEM measurements; the ECSA increases in the bimetallic PdNi/C electrocatalyst due to the smaller particle sizes and the improved dispersion of PdNi nanoparticles.

### 3.3. Performance of the Anodic Electrocatalyst for HOR

**Figure 5:**
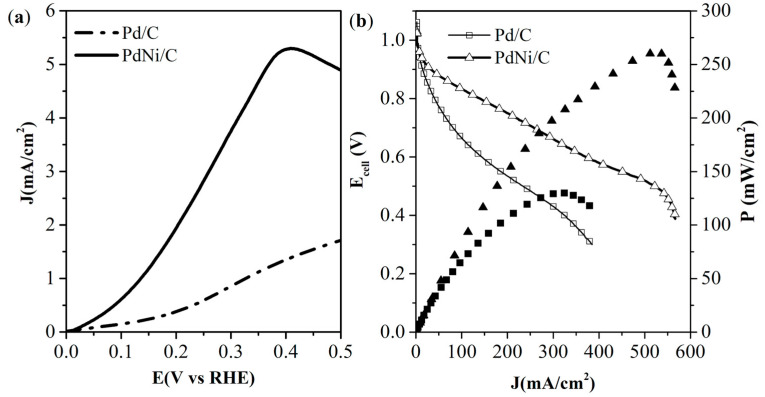
Linear scanning voltammogram at 1600 rpm for HOR on electrocatalysts in KOH 0.1 M at 10 mV s^−1^ (**a**). Polarization and power density curves of Pd/C and PdNi/C as anode in AEMFC (**b**).

**Figure 6:**
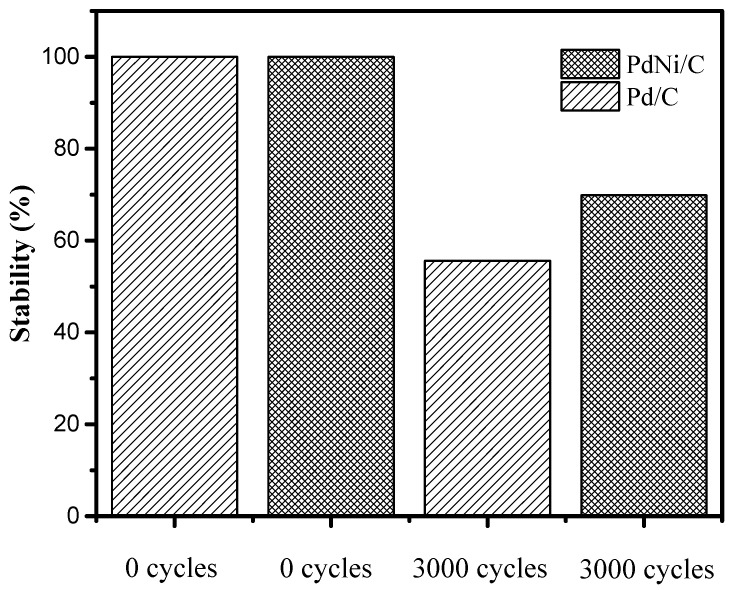
Stability tests of Pd/C and PdNi/C at 0 and 3000 cycles.

The electrochemical evaluation related to the HOR was conducted using the linear voltammetry technique under hydrodynamic conditions in a 0.1 M KOH solution. Figure 5a shows the polarization curve of Pd/C and PdNi/C electrocatalysts at a rotation speed of 1600 rpm. The current density reached by PdNi/C is higher than the value obtained by the Pd/C electrocatalyst. A displacement towards low overpotentials is evident in the polarization curve of the PdNi/C bimetallic electrocatalyst, which is related to an enhancement of the activation energy of the HOR. The stability results presented in Figure 6 for the Pd/C and PdNi/C electrocatalysts indicate that the incorporation of small amounts of nickel significantly enhances the stability of the electrocatalyst compared to Pd/C. Activity at the beginning and after 3000 cycles demonstrates this improvement: after 3000 cycles, the Pd/C electrocatalyst achieved a stability percentage of 55%, whereas the PdNi/C electrocatalyst demonstrated a stability percentage of approximately 70%.

The power density curves of the MEAs prepared with Pd/C and PdNi/C electrocatalysts in the anode gas diffusion layer of an AEMFC are shown in Figure 5b. The achieved OCV values were 1.062 V and 1.029 V for Pd/C and PdNi/C, respectively. Those values are lower than the theoretical onset potential value for the AEMFC (1.23 V). The three typical regions were clearly observed in both assemblies: the activation region, characterized by a rapid decrease in potential from the thermodynamic value; the ohmic region, where the potential gradually decreases as the current increases; and the concentration region, where water flooding creates a barrier to gas diffusion, thereby inhibiting the reactions within the AEMFC. The performance of an AEMFC with an anode based on a bimetallic PdNi/C electrocatalyst is much better than the performance obtained with a monometallic Pd/C electrocatalyst. The current density was increased 3.6 times with PdNi/C electrocatalyst than Pd/C (i.e., at 0.6 V, the obtained values were 372 mA cm^−2^ and 102 mA cm^−2^, respectively). The maximum power density obtained was twice for bimetallic PdNi/C (262 mW cm^−2^) compared to the obtained value for Pd/C (130 mW cm^−2^). This could be attributed to the faster kinetics reaction, generating a more significant number of water molecules as a product due to the presence of more hydrophilic and oxophilic sites in the electrocatalyst [35]. Table 3 presents a comparison of the electrochemical parameters of various catalysts reported in the literature. There are few reports of PdNi electrocatalysts for HOR and AEMFC studies. The bimetallic PdNi/C electrocatalyst reported shows similar current density to that reported in this work, but the mass activity (considering the mass of Pd) of our electrocatalyst exceeds the values reported by Truong [26] and slightly lower than that reported by Zitoung [16]. It should be mentioned that the conversion energy test at the AEMFC was operated at 60 °C, and the other reports declared the energy conversion test at 70 °C.

Comparing the proposed electrocatalyst with the commercial Pt/C electrocatalyst [22,36] in Table 3 shows that our bimetallic electrocatalyst exhibited a higher mass activity by more than double the mass activity of the commercial Pt/C electrocatalyst. It is noticeable that the proposed electrocatalyst exhibits better performance than the PtNi/C [22] and NiCu/KB [37] electrocatalysts.

## 4. Conclusions

In this study, low-metal-loading electrocatalysts based on bimetallic PdNi/C and monometallic Pd/C were synthesized with particle sizes smaller than 3 nm. The bimetallic PdNi/C electrocatalyst exhibited superior nanoparticle dispersion on the carbon support surface compared to the monometallic Pd/C electrocatalyst. Electrochemical characterizations revealed that the bimetallic PdNi/C electrocatalyst showed an ECSA_Pd_ more than double that of the monometallic Pd/C electrocatalyst. Similarly, PdNi/C demonstrated an ECSA_Ni_ more than 3.8 times the value achieved by Ni/C. The electrocatalytic activity in a half cell for the HOR indicated that the bimetallic PdNi/C electrocatalyst achieved higher current density and lower overpotential for HOR compared to Pd/C. Finally, MEA prepared using PdNi/C and Pd/C electrocatalyst as anode materials were tested in an AEMFC, showing that the maximum power density of PdNi/C was twice that of Pd/C. Furthermore, considering the mass activity and PdNi metals loading, the bimetallic PdNi/C electrocatalyst performance in this research surpasses the values reported by other groups.

## Figures and Tables

**Table 1:** Metallic loading, Pd:Ni ratio, and crystal size.

Catalyst	Metallic Loading (%)	Pd: Ni Ratio (w:w)	Crystal Size (Å)	BET Area (m^2^/g)
C	2.41	-	-	222.8
Pd/C	12.86	9.08:0	9.85	94.4
Ni/C	10.30	0:6.20	-	105.9
PdNi/C	23.86	12.05:6.40	12.79	70.5

**Table 2:** Summary of ECSA of prepared electrocatalysts.

Electrocatalyst	ECSA_Ni_ (cm^2^ mg^−1^)	ECSA_CO_-_Pd_ (m^2^ g^−1^)	ECSA_H_-_Pd_ (cm^2^ mg^−1^)
Ni/C	4.87	-	-
Pd/C	-	11.88	13.93
PdNi/C	18.67	23.98	22.83

**Table 3:** Comparison of AEMFC performance of different materials as anodes.

Anode	OCP (V)	J_0.6 V_ (mA cm^−2^)	P_max_ (mW cm^−2^)	MA (mW mg^−1^_Pd or Pt_)	Reference
Pd/C	1.06	102	130	260	This work
PdNi/C	1.03	372	262	762	This work
Pd/C ^a^	1.01	80	180	130	16
Pd@Ni ^a^	1.04	400	400	1300	16
PdNi/C ^b^	0.96	302	302	403	26
PtNi/C ^c^	1.03	253	151	652	27
Pt/C _(Commercial)_	1.05	258	163	325	27
Pt/C _(Commercial)_	1.07	250	109	218	36
NiCu/KB ^d^	1.00	110	78	---	37

^a^: The wt.% ratio of Pd to Ni was 20:80, and the active electrode area was 5 cm^2^. ^b^: The wt.% ratio of Pd to Ni was 50:50, and the active electrode area was 10.24 cm^2^. ^c^: The wt.% ratio of Pt to Ni was 57:43, and the active electrode area was 5 cm^2^. ^d^: The wt.% ratio of Ni to Cu was 95:5, and the active electrode area was 5 cm^2^.

## Data Availability

The data presented in this study are available from the corresponding author.

## Supplementary Materials

The following supporting information can be downloaded at: https://www.mdpi.com/article/10.3390/nano15090664/s1, Figure S1. BET analysis of of C, Ni/C and PdNi/C materials. Figure S2. SEM image and EDS mapping of PdNi/C materials.
