# Supplementary material for: Electrocatalyst of PdNi Particles on Carbon Black for Hydrogen Oxidation Reaction in Alkaline Membrane Fuel Cell
Source: Nanomaterials (Basel). 2025 Apr 27;15(9):664. doi: 10.3390/nano15090664 (PMC12073287; doi:10.3390/nano15090664)
Supplement: Supplementary file 1 [file nanomaterials-15-00664-s001.zip › nanomaterials-3550030-supplementary.pdf]

Electrocatalyst of PdNi particles on carbon black for hydrogen oxidation reaction in alkaline membrane fuel cell

Carolina Silva-Carrillo,[1,2] Edgar Alonso Reynoso-Soto,[1] Ivan Cruz-Reyes,[1] Moisés Israel Salazar-Gastélum,[1] Balter Trujillo-Navarrete,[1] Sergio Pérez-Sicairos,[1] José Roberto Flores-Hernández,[3] Tatiana Romero-Castañón,[3] Francisco Paraguay-Delgado,[4] Rosa María Félix-Navarro[1,*]

Reagents.

All chemicals used in this study were utilized without further purification. Potassium hexachloropalladate (IV) (K_2PdCl_6, 98%), nickel (II) acetate tetrahydrate ($Ni(OCOCH_3)_2 \cdot 4H_2O$, 98%), hexadecyltrimethylammonium bromide (CTAB, 99%), sodium borohydride ($NaBH_4$, 99%), sodium citrate tribasic hydrate ($C_6H_5Na_3O_7 \cdot xH_2O$, 99%), potassium hydroxide (KOH, 99.9%), and a 5% Nafion®-117 solution were sourced from Sigma-Aldrich®. Nitric acid (HNO_3, 70%), 2-propanol (C_3H_8O, 99%), and methanol (CH_3OH, 99.9%) were obtained from Fermont. Pt/C (20%) and Carbon black (Vulcan XC-72) were supplied by Fuel Cell Store®. Hydrogen (H_2, 99%), argon (Ar, 99%), nitrogen (N_2, 99%), and carbon monoxide (CO, 99%) were acquired through INFRA. All aqueous solutions were prepared with Milli-Q water (18 MΩ, Thermo Scientific®). The AEMION+® AF1-HNN8-25 membrane, with a thickness of 50 μm, along with AEMION+® inomer825, was procured from Ionomr Innovations Inc.

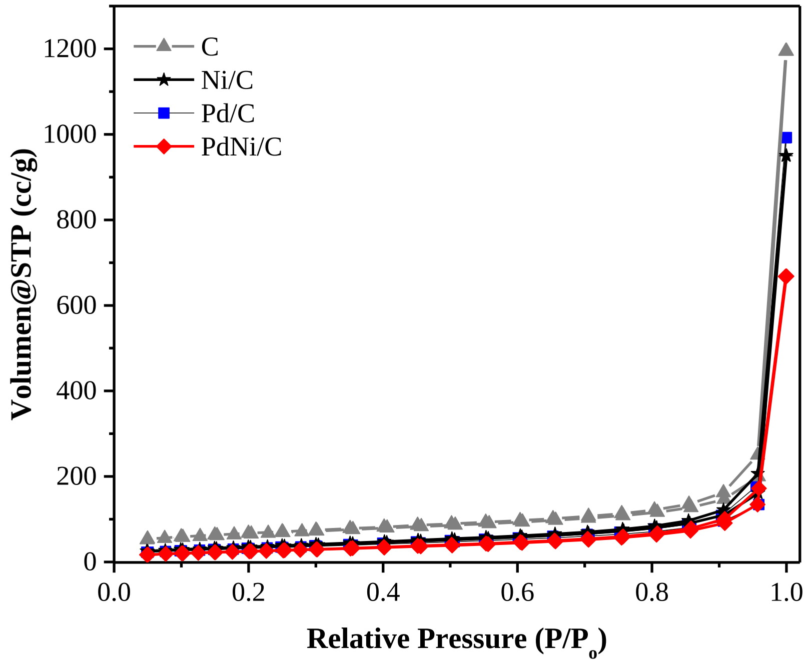

Figure S1. BET analysis of of C, Ni/C and PdNi/C materials.

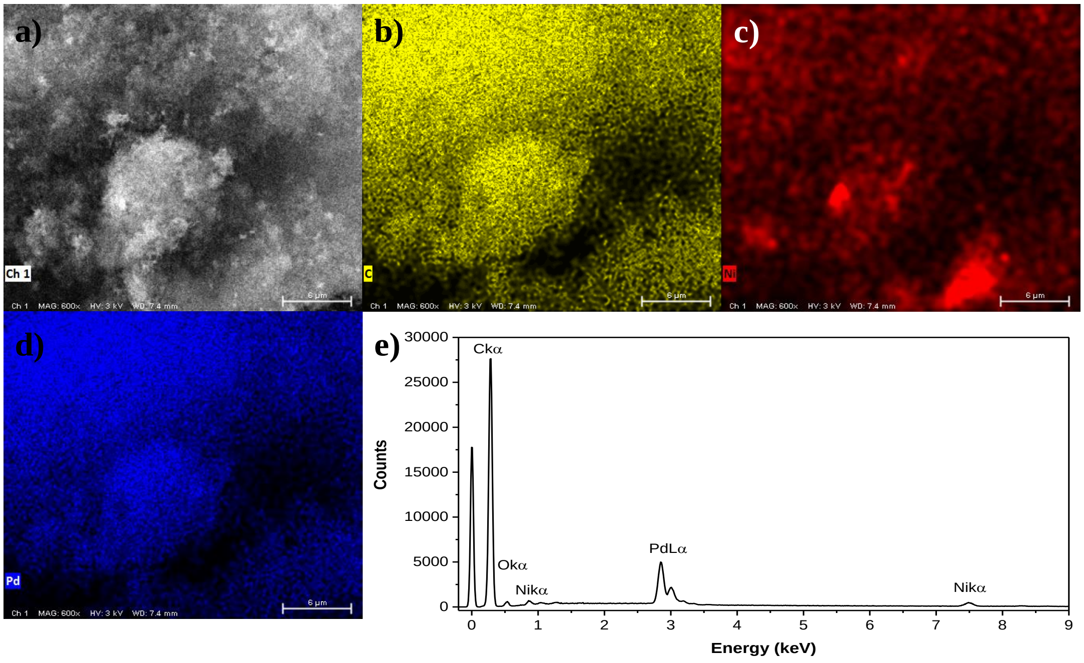

Figure S2. SEM image and EDS mapping of PdNi/C materials.
